# *N*,*N*′-Bis[(*E*)-(5-chloro-2-thienyl)methyl­idene]ethane-1,2-diamine

**DOI:** 10.1107/S160053681004167X

**Published:** 2010-10-23

**Authors:** R. Prasath, P. Bhavana, Seik Weng Ng, Edward R. T. Tiekink

**Affiliations:** a Chemistry Group, BITS, Pilani – K. K. Birla Goa Campus, Goa, India 403 726; b Department of Chemistry, University of Malaya, 50603 Kuala Lumpur, Malaysia

## Abstract

The full mol­ecule of the title compound, C_12_H_10_Cl_2_N_2_S_2_, is generated by the application of a centre of inversion. The thio­phene and imine residues are co-planar [the N—C—C—S torsion angle is −2.5 (4)°] and the conformation about the imine bond [1.268 (4) Å] is *E*. Supra­molecular arrays are formed in the *bc* plane *via* C—Cl⋯π inter­actions and these stack along the *a* axis.

## Related literature

For background to 2-substituted thio­phenes, see: Campaigne (1984 ▶); Kleemann *et al.* (2006 ▶). For related structures, see: Wang *et al.* (2007 ▶); Wardell *et al.* (2010 ▶); Prasath *et al.* (2010 ▶).
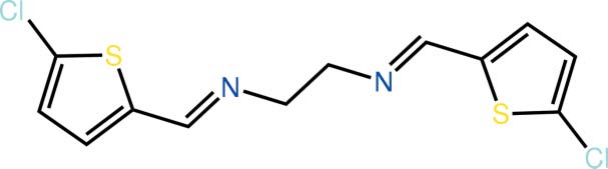


## Experimental

### 

#### Crystal data

C_12_H_10_Cl_2_N_2_S_2_
*M* *_r_* = 317.24
Monoclinic, 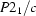

*a* = 14.682 (2) Å
*b* = 4.7016 (7) Å
*c* = 10.6607 (15) Å
β = 109.439 (2)°
*V* = 693.92 (17) Å^3^
*Z* = 2
Mo *K*α radiation
μ = 0.75 mm^−1^
*T* = 100 K
0.25 × 0.15 × 0.05 mm

#### Data collection

Bruker SMART APEX diffractometer
Absorption correction: multi-scan (*SADABS*; Sheldrick, 1996 ▶) *T*_min_ = 0.835, *T*_max_ = 0.963
5721 measured reflections
1576 independent reflections
1270 reflections with *I* > 2σ(*I*)
*R*_int_ = 0.058

#### Refinement

*R*[*F* ^2^ > 2σ(*F* ^2^)] = 0.038
*wR*(*F* ^2^) = 0.117
*S* = 1.01
1576 reflections
82 parameters
H-atom parameters constrained
Δρ_max_ = 0.36 e Å^−3^
Δρ_min_ = −0.48 e Å^−3^

### 

Data collection: *APEX2* (Bruker, 2008 ▶); cell refinement: *SAINT* (Bruker, 2008 ▶); data reduction: *SAINT*; program(s) used to solve structure: *SHELXS97* (Sheldrick, 2008 ▶); program(s) used to refine structure: *SHELXL97* (Sheldrick, 2008 ▶); molecular graphics: *ORTEP-3* (Farrugia, 1997 ▶) and *DIAMOND* (Brandenburg, 2006 ▶); software used to prepare material for publication: *publCIF* (Westrip, 2010 ▶).

## Supplementary Material

Crystal structure: contains datablocks global, I. DOI: 10.1107/S160053681004167X/hg2729sup1.cif

Structure factors: contains datablocks I. DOI: 10.1107/S160053681004167X/hg2729Isup2.hkl

Additional supplementary materials:  crystallographic information; 3D view; checkCIF report

## Figures and Tables

**Table 1:** Hydrogen-bond geometry (Å, °) *Cg*1 is the centroid of the S3,C3–C6 ring.

*D*—H⋯*A*	*D*—H	H⋯*A*	*D*⋯*A*	*D*—H⋯*A*
C6—Cl1⋯*Cg*1^i^	1.71 (1)	3.52 (1)	3.994 (3)	93 (1)

Symmetry code: (i) 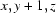.

**Fig. 1.**
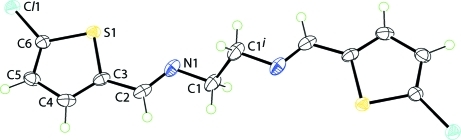
Molecular structure of (I) showing the atom-labelling scheme and displacement ellipsoids at the 50% probability level. Symmetry operation i: 1 - x, 1 - y, 1 - z.

**Fig. 2.**
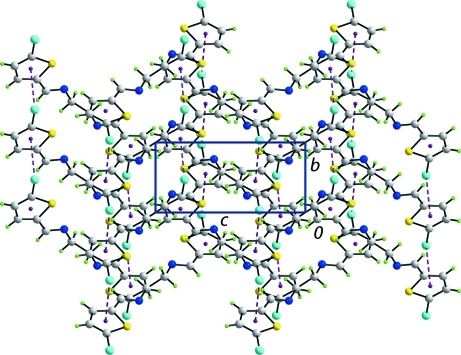
A view of the supramolecular 2-D array in the bc plane mediated by C—Cl···π interactions (purple dashed lines).

**Fig. 3.**
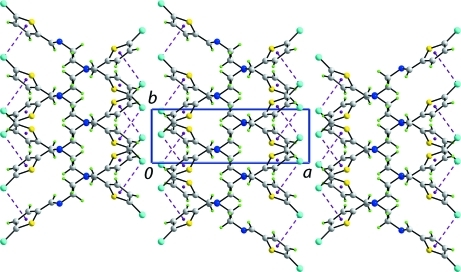
Stacking of layers along the a axis in the crystal structure of (I). The C—Cl···π interactions are shown as purple dashed lines.

## supplementary crystallographic information

### Comment

Interest in their putative biological activity (Wardell *et al.*, 2010) motivates studies of 2-substituted thiophene rings (Campaigne, 1984; Kleemann *et al.*, 2006), including on-going crystallographic investigations (Wardell *et al.* 2010; Prasath *et al.*, 2010).

The asymmetric unit of (I), Fig. 1, comprises half a molecule with the full molecule generated by a crystallographic centre of inversion. The thiophene residue is co-planar with the imine group as seen in the value of the N1—C2—C3—S1 torsion angle of -2.5 (4) °. The conformation about the imine N1—C2 [1.268 (4) Å] bond is *E*. The observed conformation matches closely those found for related compounds (Wang *et al.*, 2007; Prasath *et al.*, 2010).

The most prominent contacts in the crystal packing are of the type C—Cl···π, Table 1. These serve to connect molecules into a 2-D array in the *bc* plane, Fig. 2, which stack along the *a* axis, Fig. 3, with the chlorido atoms facing each other. Concerning the latter, the closest interlayer Cl···Cl contact is 3.3831 (11) Å [symmetry operation: 2 - *x*, -1/2 + *y*, 3/2 - *z*].

### Experimental

A mixture of 5-chloro-2-thiophenecarboxaldehyde (0.43 ml, 0.004 *M*) and ethylenediamine (0.13 ml, 0.002 *M*) was stirred in dichloromethane for 3 h at room temperature. The solvent from the reaction mixture was removed under reduced pressure. The resulting solid was dried and purified by column chromatography using a 1:3 mixture of ethyl acetate and hexane. Recrystallization was by slow evaporation of a dichloromethane solution of (I) which yielded colourless needles (yield: 73%). *M*. pt. 353–355 K.

### Refinement

Carbon-bound H-atoms were placed in calculated positions (C—H 0.95 to 0.99 Å) and were included in the refinement in the riding model approximation, with *U*_iso_(H) set to 1.2*U*_equiv_(C). In the final refinement a low angle reflection evidently effected by the beam stop was omitted, *i.e*. (100).

### Crystal data

**Table:**

C_12_H_10_Cl_2_N_2_S_2_	*F*(000) = 324
*M**_r_* = 317.24	*D*_x_ = 1.518 Mg m^−^^3^
Monoclinic, *P*2_1_/*c*	Mo *K*α radiation, λ = 0.71073 Å
Hall symbol: -P 2ybc	Cell parameters from 2363 reflections
*a* = 14.682 (2) Å	θ = 3.0–28.0°
*b* = 4.7016 (7) Å	µ = 0.75 mm^−^^1^
*c* = 10.6607 (15) Å	*T* = 100 K
β = 109.439 (2)°	Prism, colourless
*V* = 693.92 (17) Å^3^	0.25 × 0.15 × 0.05 mm
*Z* = 2	

### Data collection

**Table:**

Bruker SMART APEX diffractometer	1576 independent reflections
Radiation source: fine-focus sealed tube	1270 reflections with *I* > 2σ(*I*)
graphite	*R*_int_ = 0.058
ω scans	θ_max_ = 27.5°, θ_min_ = 2.9°
Absorption correction: multi-scan (*SADABS*; Sheldrick, 1996)	*h* = −18→18
*T*_min_ = 0.835, *T*_max_ = 0.963	*k* = −6→6
5721 measured reflections	*l* = −13→13

### Refinement

**Table:**

Refinement on *F*^2^	Primary atom site location: structure-invariant direct methods
Least-squares matrix: full	Secondary atom site location: difference Fourier map
*R*[*F*^2^ > 2σ(*F*^2^)] = 0.038	Hydrogen site location: inferred from neighbouring sites
*wR*(*F*^2^) = 0.117	H-atom parameters constrained
*S* = 1.01	*w* = 1/[σ^2^(*F*_o_^2^) + (0.0662*P*)^2^ + 0.3727*P*] where *P* = (*F*_o_^2^ + 2*F*_c_^2^)/3
1576 reflections	(Δ/σ)_max_ = 0.001
82 parameters	Δρ_max_ = 0.36 e Å^−^^3^
0 restraints	Δρ_min_ = −0.48 e Å^−^^3^

### Special details

**Table:**

Geometry. All e.s.d.'s (except the e.s.d. in the dihedral angle between two l.s. planes) are estimated using the full covariance matrix. The cell e.s.d.'s are taken into account individually in the estimation of e.s.d.'s in distances, angles and torsion angles; correlations between e.s.d.'s in cell parameters are only used when they are defined by crystal symmetry. An approximate (isotropic) treatment of cell e.s.d.'s is used for estimating e.s.d.'s involving l.s. planes.
Refinement. Refinement of *F*^2^ against ALL reflections. The weighted *R*-factor *wR* and goodness of fit *S* are based on *F*^2^, conventional *R*-factors *R* are based on *F*, with *F* set to zero for negative *F*^2^. The threshold expression of *F*^2^ > σ(*F*^2^) is used only for calculating *R*-factors(gt) *etc*. and is not relevant to the choice of reflections for refinement. *R*-factors based on *F*^2^ are statistically about twice as large as those based on *F*, and *R*- factors based on ALL data will be even larger.

### Fractional atomic coordinates and isotropic or equivalent isotropic displacement parameters (Å^2^)

**Table:**

	*x*	*y*	*z*	*U*_iso_*/*U*_eq_	
Cl1	0.94014 (5)	1.46904 (14)	0.80609 (6)	0.0258 (2)	
S1	0.76180 (5)	1.12742 (14)	0.69149 (6)	0.0220 (2)	
N1	0.58522 (16)	0.7601 (5)	0.6104 (2)	0.0286 (5)	
C1	0.4988 (2)	0.5840 (6)	0.5603 (3)	0.0333 (7)	
H1A	0.4406	0.7062	0.5355	0.040*	
H1B	0.4955	0.4514	0.6308	0.040*	
C2	0.63515 (19)	0.7367 (6)	0.7325 (3)	0.0267 (6)	
H2	0.6151	0.6077	0.7868	0.032*	
C3	0.72211 (19)	0.9026 (5)	0.7907 (3)	0.0238 (6)	
C4	0.7822 (2)	0.9074 (6)	0.9193 (3)	0.0282 (6)	
H4	0.7716	0.7970	0.9879	0.034*	
C5	0.8620 (2)	1.0934 (6)	0.9411 (3)	0.0271 (6)	
H5	0.9109	1.1220	1.0247	0.033*	
C6	0.85924 (19)	1.2254 (6)	0.8263 (2)	0.0220 (5)	

### Atomic displacement parameters (Å^2^)

**Table:**

	*U*^11^	*U*^22^	*U*^33^	*U*^12^	*U*^13^	*U*^23^
Cl1	0.0284 (4)	0.0242 (4)	0.0285 (3)	−0.0067 (2)	0.0141 (3)	−0.0066 (2)
S1	0.0249 (4)	0.0199 (4)	0.0230 (3)	−0.0031 (2)	0.0104 (3)	−0.0007 (2)
N1	0.0241 (12)	0.0212 (12)	0.0432 (14)	−0.0047 (9)	0.0149 (10)	−0.0032 (10)
C1	0.0288 (15)	0.0285 (15)	0.0463 (18)	−0.0089 (12)	0.0173 (13)	−0.0043 (13)
C2	0.0302 (14)	0.0184 (13)	0.0402 (16)	−0.0028 (11)	0.0232 (12)	−0.0029 (11)
C3	0.0287 (14)	0.0169 (13)	0.0323 (14)	−0.0025 (10)	0.0187 (12)	−0.0013 (10)
C4	0.0416 (17)	0.0220 (14)	0.0274 (13)	−0.0028 (12)	0.0202 (12)	0.0001 (10)
C5	0.0353 (15)	0.0261 (15)	0.0219 (12)	−0.0025 (11)	0.0122 (11)	−0.0033 (10)
C6	0.0259 (13)	0.0188 (13)	0.0241 (12)	−0.0002 (10)	0.0122 (10)	−0.0050 (9)

### Geometric parameters (Å, °)

**Table:**

Cl1—C6	1.714 (3)	C2—C3	1.448 (4)
S1—C6	1.719 (3)	C2—H2	0.9500
S1—C3	1.728 (3)	C3—C4	1.362 (4)
N1—C2	1.268 (4)	C4—C5	1.419 (4)
N1—C1	1.459 (3)	C4—H4	0.9500
C1—C1^i^	1.519 (6)	C5—C6	1.361 (4)
C1—H1A	0.9900	C5—H5	0.9500
C1—H1B	0.9900		
			
C6—S1—C3	90.50 (13)	C4—C3—S1	111.7 (2)
C2—N1—C1	117.5 (2)	C2—C3—S1	119.7 (2)
N1—C1—C1^i^	110.1 (3)	C3—C4—C5	113.4 (2)
N1—C1—H1A	109.6	C3—C4—H4	123.3
C1^i^—C1—H1A	109.6	C5—C4—H4	123.3
N1—C1—H1B	109.6	C6—C5—C4	111.0 (3)
C1^i^—C1—H1B	109.6	C6—C5—H5	124.5
H1A—C1—H1B	108.2	C4—C5—H5	124.5
N1—C2—C3	121.3 (2)	C5—C6—Cl1	127.1 (2)
N1—C2—H2	119.4	C5—C6—S1	113.4 (2)
C3—C2—H2	119.4	Cl1—C6—S1	119.53 (15)
C4—C3—C2	128.6 (2)		
			
C2—N1—C1—C1^i^	−126.6 (3)	S1—C3—C4—C5	0.0 (3)
C1—N1—C2—C3	179.9 (2)	C3—C4—C5—C6	0.2 (4)
N1—C2—C3—C4	178.4 (3)	C4—C5—C6—Cl1	179.4 (2)
N1—C2—C3—S1	−2.5 (4)	C4—C5—C6—S1	−0.3 (3)
C6—S1—C3—C4	−0.1 (2)	C3—S1—C6—C5	0.3 (2)
C6—S1—C3—C2	−179.4 (2)	C3—S1—C6—Cl1	−179.49 (17)
C2—C3—C4—C5	179.1 (3)		

Symmetry codes: (i) −*x*+1, −*y*+1, −*z*+1.

### Hydrogen-bond geometry (Å, °)

**Table:**

Cg1 is the centroid of the S3,C3–C6 ring.

**Table:**

*D*—H···*A*	*D*—H	H···*A*	*D*···*A*	*D*—H···*A*
C6—Cl1···Cg1^ii^	1.714 (3)	3.5182 (14)	3.994 (3)	93.01 (10)

Symmetry codes: (ii) *x*, *y*+1, *z*.
